# Praziquantel ameliorates leflunomide-induced nephrotoxicity in mice: a novel therapeutic approach targeting TGF-β/Smad/Wnt/β-catenin/NF-κB/PPAR-γ signaling and Nrf-2

**DOI:** 10.1007/s00210-025-04920-3

**Published:** 2026-01-10

**Authors:** Weam A. Elkady, Safaa A. Faheem, Reem M. Hazem, Naglaa F. El-Orabi

**Affiliations:** 1 Department of Pharmacology and Toxicology, Faculty of Pharmacy, Egyptian Russian University, Cairo-Suez Road, Badr City, 11829 Cairo Egypt; 2 Department of Pharmacology and Toxicology, Faculty of Pharmacy, Suez Canal University, Ismailia, 41522 Egypt

**Keywords:** Leflunomide, Nephrotoxicity, Praziquantel, TGF-β/SMAD signaling, Wnt/β-catenin signaling, PPAR-γ

## Abstract

Leflunomide (LF), an essential immunomodulator for autoimmune disorders, poses a risk of nephrotoxicity. This work was designed to examine the possible mechanisms of LF nephrotoxicity and to investigate the potential protective role of praziquantel (PZQ), an anti-helminthic drug, against LF-induced nephrotoxicity in mice. Forty male albino mice were randomly allocated into four groups. Group one acted as the control. Group two received LF at (10 mg/kg, P.O.). The third group was given both LF (10 mg/kg, P.O.) and PZQ (300 mg/kg, P.O.). Finally, the fourth group was given PZQ (300 mg/kg, P.O.). After eight weeks, the mice were euthanized following anesthesia, and their kidneys were extracted for biochemical and histopathological analysis. Leflunomide (LF) significantly increased serum creatinine (0.88 ± 0.01 mg/dL vs. 0.16 ± 0.01 mg/dL, *p* < 0.05) and BUN levels (69.15 ± 3.73 mg/dL vs. 32.52 ± 1.18 mg/dL, *p* < 0.05), while reducing albumin levels by 27.26%. Co-treatment with praziquantel (PZQ) markedly restored renal function, decreasing creatinine and BUN by 71.6% and 34.2%, respectively (*p* < 0.05), and increasing albumin by 1.2-fold. PZQ also significantly reduced oxidative stress markers (MDA 56.98%, *p* < 0.05) and pro-inflammatory cytokines (IL-6 − 73.69%, *p* < 0.05), while upregulating antioxidant (Nrf-2 + 6.7-fold) and anti-inflammatory (PPAR-γ + 3.1-fold) pathways. These statistically significant improvements confirm the nephroprotective efficacy of PZQ against LF-induced nephrotoxicity through modulation of TGF-β/Smad, Wnt/β-catenin, NF-κB, Nrf-2, and PPAR-γ signaling pathways.

## Introduction

Leflunomide (LF), a synthetic isoxazole derivative, was introduced in late 1998 as a member of a new class of inhibitors targeting dihydroorotate dehydrogenase (DHODH), an enzyme critical for the de novo biosynthesis of pyrimidine nucleotides (Zheng et al. 2023). By non-competitive suppression of pyrimidine synthesis, LF effectively inhibits the proliferation of mitogen-stimulated lymphocytes (Alamri et al. 2021; Jiang et al. 2024). Due to its specific mechanism of action, LF has been approved as an alternative treatment option for patients with refractory rheumatoid arthritis. The FDA recommended precautionary liver function monitoring during LF therapy in the early 2000 s (Alfaro-Lara et al. 2019). In 2010, a black box warning was issued as a result of the potential for severe liver injury (Elshaer et al. 2019). Recently, LF was shown to induce dose-dependent renal injury, characterized by leukocyte infiltration, glomerular and tubular degeneration, and fibrosis (Aljohani et al. 2022).

Nephrotoxicity is a rapid deterioration of renal function caused by the harmful effects of pharmaceuticals or chemicals, such as immunosuppressive agents, frequently employed in treating various diseases (Al-Naimi et al. 2019; Wu and Huang 2018). It occurs by multiple mechanisms involving growth factors, cytokines, toxins, and stress chemicals, which activate particular signaling pathways (Rivera 2022).

Transforming growth factor-β (TGF-β) is a pivotal cytokine in nephrotoxicity, vital for normal development and tissue regeneration; however, its overexpression significantly contributes to renal injury (Meng et al. 2016). In the canonical Smad-dependent TGF-β signaling pathway, Smad proteins are classified as receptor-regulated Smads (Smad2–Smad3), common mediator Smads (Smad4), and inhibitory Smads (Smad7). Smad3 induces pro-fibrotic effects, whereas Smad2 and Smad7 confer renal protection (Cutroneo and Phan 2003; Lan 2012). Smad4 demonstrates dual functions, amplifying Smad3-induced fibrosis and mitigating inflammation (Lan 2011). In pathological conditions, the expression of Smad2 and Smad3 is elevated, whereas the expression of Smad7 is reduced, enhancing pro-fibrotic signaling (Chen et al. 2018).

Transforming growth factor-β interacts with various signaling pathways, including Wnt/β-catenin and Peroxisome proliferator-activated receptor gamma (PPAR-γ), significantly affecting the advancement of nephrotoxicity (Meng et al. 2016; Kökény et al. 2021). The Wnt/β-catenin signaling system is essential for human health, as it governs embryogenesis by coordinating tissue organization and maintaining tissue integrity throughout life. However, dysregulation of Wnt/β-catenin signaling is implicated in various clinical disorders (Faheem et al. 2022), including renal damage (Feng et al. 2018). β-catenin serves as both a structural protein and a transcription factor, with its function being influenced by Wnt ligand presence and its cellular localization (Huffstater et al. 2020), thereby promoting Wnt-dependent gene expression, including c-myc and cyclin D1(Schunk et al. 2021).

The interaction between the Wnt/β-catenin and TGF-β/Smad pathways in kidney damage is multifaceted. Components of the Wnt pathway, such as the Wnt3a ligand and the c-myc target gene, can stimulate the TGF-β/Smad pathway. At the same time, TGF-β simultaneously augments Wnt/β-catenin signaling by interacting with the E3 ubiquitin ligase Beta-transducin repeats-containing proteins (β-TrCP), which suppresses the ubiquitination of β-catenin and facilitates its stability. This demonstrates their interaction’s context-dependent and dynamically evolving nature (Askari 2017; Vallée et al. 2017; Dao et al. 2007).

Peroxisome proliferator-activated receptor gamma, a nuclear hormone receptor superfamily member, was initially recognized in adipocytes and is considered a key regulator of adipogenesis. PPAR-γ is essential for regulating various cell functions, such as proliferation, apoptosis, and differentiation, as well as inflammation, angiogenesis, and immune response processes (Zheng et al. 2002). It also exhibits anti-inflammatory and anti-oxidant effects by modulating the NLRP3 inflammasome, one of the most extensively studied inflammasome complexes, reducing superoxide production and alleviating mitochondrial dysfunction (Li et al. 2023; Sharma and Patial 2022). Several inflammatory cytokines and intracellular signaling pathways, such as TGF-β1, the canonical Wnt/β-catenin pathway and NFκB signaling, reduce PPAR-γ expression. TGF-β1 interacts with the Wnt/β-catenin signaling cascade, typically producing effects that oppose those of PPAR-γ in various pathologies. In liver fibrosis, the TGF-β/Smad and canonical Wnt pathways mutually stimulate each other and downregulate PPAR-γ expression at the transcriptional level. Conversely, PPAR-γ agonists activate Smad7, Glycogen synthase kinase-3 beta (GSK-3β), Dickkopf-1 (DKK) and IκB which inhibit TGF-β, Wnt/β-catenin and NFκB signaling (Vallée et al. 2017; Lakshmi et al. 2017).

Praziquantel (PZQ), a schistosomicide that has been in use for more than three decades, is prominent for its safety, efficacy, and mild adverse effects. Long-term use may induce antifibrotic effects in liver disease, according to previous studies. The underlying mechanism involves the induction of Smad7 expression, which blocks the TGF-β/Smad signaling pathway and subsequently lowers the transcription of pro-fibrotic genes. (Liu et al. 2019; Niu et al. 2022).

From this point, this study subsequently explored the possible nephroprotective effects of PZQ as a Smad7 inducer against LF-induced nephrotoxicity in mice. Specifically, it examines whether PZQ can activate Smad7 and modulate the TGF-β/Smad/Wnt/β-catenin/NF-κB/Nrf-2 and PPAR-γ signaling pathways to reduce kidney damage.

## Materials and methods

### Drugs and chemicals

Leflunomide, PZQ, and carboxymethyl cellulose were supplied by Sigma-Aldrich (St. Louis, MO, USA) following a purchase request.

### Animals

Forty male Swiss albino mice, 6 weeks of age, weighing between 20 and 30 g, were procured from VACCERA (Egypt’s Holding Company for Biological Products and Vaccines). The animals were in stainless steel cages lined with hardwood bedding under controlled laboratory conditions (25 ± 1 °C; 12-h light/dark cycle). The mice had free access to food and water and were fed a standard rodent pellet diet (El-Nasr Company, Abou-Zaabal, Cairo, Egypt) containing 20% protein, 5% fat, 5% fiber, 55% carbohydrates, and essential vitamins and minerals. Before the start of the experiment, the mice were allowed to acclimate to the laboratory conditions for one week. All experimental procedures were conducted in accordance with the standards of Suez Canal University’s Research Ethics Committee (protocol serial number 202311MA2).

### Experimental design

Forty male mice were allocated at random into four groups of ten. Group 1, the control group, received 1% CMC (10 ml/kg every 48 h, p.o.) for eight weeks. Group 2, the LF group, received LF (10 mg/kg/every 48 h, p.o.) (Aljohani et al. 2022) for eight weeks. Group 3, the LF/PZQ group, received LF (10 mg/kg every 48 h, p.o.) and PZQ (300 mg/kg/day, p.o.) (Liu et al. 2019) for eight weeks. Group 4, the PZQ group, received PZQ (300 mg/kg/day, p.o.) simultaneously.

### Sample collection

At the conclusion of the experiment, blood samples were collected from the orbital sinus of mice under anesthesia induced by thiopental sodium (50 mg/kg) (Hazem et al. 2022). The collected blood was centrifuged to obtain serum, which was stored at − 80 °C for subsequent biochemical and ELISA assays. Each experimental group consisted of ten mice: six were used for biochemical and ELISA analyses, while from the remaining four, the right kidneys were fixed in 10% buffered formalin for histopathological and immunohistochemical examinations. The left kidneys from three of these animals were preserved at − 80 °C for molecular (RT-qPCR) analysis.

### Colorimetric assessment of serum creatinine, urea, and albumin

Serum creatinine was measured utilizing a commercial assay kit (Cat. No. CR1250; Biodiagnostics, Giza, Egypt), blood urea nitrogen (BUN) was determined using a kit (Cat. No. EIABUN; Thermo Fisher Scientific, MA, USA), and albumin levels were assessed with a kit (Catalog No. AB1010; Biodiagnostics, Giza, Egypt). All procedures followed the manufacturer’s instructions, with results expressed in mg/dL for creatinine and BUN, and in g/dL for albumin.

### Histopathological examination

Renal tissue specimens were preserved in 10% neutral-buffered formalin for 72 h, dehydrated in ethanol, cleansed with xylene, and embedded in paraffin wax. The rotary microtome was employed to produce sections of 4 μm thickness, which were then affixed to glass slides, stained with Hematoxylin and Eosin following the Bancroft et al. protocol, and analyzed under a light microscope for morphological characterization (Suvarna et al. 2012).

### Enzyme-linked immunosorbent assay (ELISA)

#### Assessment of oxidative stress and anti-oxidant parameters: MDA, CAT, SOD, GSH, Nrf-2, and HO-1

Renal malondialdehyde (MDA) levels were quantified with a commercial ELISA kit (Cat. No: MBS269473, MyBioSource, San Diego, California, USA) following the manufacturer’s guidelines. The anti-oxidants activity was evaluated by quantifying catalase (CAT), superoxide dismutase (SOD) and glutathione (GSH) activities and Nuclear factor erythroid 2-related factor 2 (Nrf-2) utilizing ELISA kits (Cat. No: MBS9307246, MBS265351, MBS267424 and MBS2516218, respectively, MyBioSource, San Diego, California, USA) in accordance with the manufacturer’s instructions. Meanwhile, heme oxygenase 1 (HO-1) (Cat. No: E4524-100, Biovision, Milpitas, CA, USA). The results of MDA and GSH were expressed as nmol/mg protein. While the results of CAT and SOD were expressed as u/mg protein. Finally, the results of Nrf-2 and HO-1 were expressed as ng/mg protein.

#### Assessment of TNF-α, IL-1 β, IL-6, NF-κB, and PPAR-γ

The levels of tumor necrosis factor-alpha (TNF-α), interleukin-1β (IL-1β), interleukin-6 (IL-6), nuclear factor kappa B (NF-κB), and PPAR-γ in kidney homogenates were measured, adhering to the manufacturer’s instructions. TNF-α, IL-6, and IL-1β were quantified in pg/mg of protein, with TNF-α and IL-6 sourced from Cusabio (Cat no. CSB-E04741m and CSB-E04639m, respectively, Wuhan, China) and IL-1β from Mybiosource (Cat no. MBS175967, San Diego, CA, USA). PPAR-γ and NF-κB were measured in ng/mg, obtained from MyBioSource (Cat no. MBS2501353 and MBS2708370, respectively, San Diego, CA, USA).

### Real-time polymerase chain reaction for Wnt/β-catenin and TGF-β signaling genes expression

Kidney tissue was homogenized, and total RNA for Wnt3a, β-catenin, FZD7, c-Myc, Smad2, Smad3, and Smad7 analysis was extracted using the RNeasy Mini Kit (Qiagen, Venlo, Netherlands). RNA purity was assessed spectrophotometrically by the A260/280 ratio. One microgram of RNA was reverse-transcribed to cDNA with the Reverse Transcription System (Promega, Leiden, Netherlands) following the manufacturer’s instructions. Quantitative RT-PCR for Wnt3a, β-catenin, FZD7, c-Myc, Smad2, Smad3, and Smad7 was performed using SYBR Green Master Mix (Applied Biosystems, CA, USA). Each 25 µL reaction contained 5 µL cDNA, 12.5 µL SYBR Green master mix, 5.5 µL RNase-free water, and 2 µL of each primer. Amplification consisted of 40 cycles of 95 °C for 15 s (denaturation), 60 °C for 60 s (annealing), and 72 °C for 60 s (extension). Relative gene expression was calculated by the 2^ − ΔΔCt method after normalization to β-actin (Livak and Schmittgen 2001). The primer sequences used for parameter amplification are detailed in Table 1**.**

**Table 1:** Primer used for RT-qPCR analysis

mRNA species	Accession Number	Primer sequence (5'–3')
*β-actin*	NM-007393.5	F: GAGACCTTCAACACCCCAGC R: ATGTCACGCACGATTTCCC
*Smad2*	NM_010754	F: AGAGAGTTGAGACACCAGTTTTGC R: ATAGTCATCCAG AGGCGGAAGTT
*Smad3*	NM_016769	F: CTCTCCAATGTCAACAGGAATG R: AACTGGTAGACAGCCTCAAAGC
*Smad7*	Mm00484742_m1	F: AGAGGCTGTGTTGCTGTGAATC R: GCAGAGTCGGCTAAGGTGATG
*Wnt3a*	NM_009522	F: TCTGCAGGAACTACGTGGAGATCA R: TCCCGAGAGACCATTCCTCCAAAT
*β -Catenin*	MP200631	F: TTCTGGTGCCACTACCACAGC R: TGCATGCCCTCATCTAATGTC
*FZD7*	NM_008057	F: CCGTACCACGGAGAGAAGG R: GCGGAGTTCGGGAGAACAC
*c-myc*	NM_001177354	F: GTCTTCCCCTACCCGCTC R: CTGTCCAACTTGGCCCTC

### Immunohistochemistry

Serial sections were subjected to deparaffinization, hydration, and antigen retrieval using EDTA (pH 8). TGF-β1 antibody (GTX45121, 1:200, GeneTex, Irvine, CA, USA) was applied to slides after 12 min of incubation with 0.3% hydrogen peroxide and maintained at 4 °C for 12 h. Secondary antibodies were administered for an hour following three phosphate-buffered saline washes. Mayer’s hematoxylin was employed as a counterstain, and the color development was achieved using Power-Stain™ 1.0 Poly HRP with 3,3'-diaminobenzidine (Genemed Biotechnologies, South San Francisco, CA, USA). Under a light microscope, the stained sections were evaluated and quantified using ImageJ (NIH, Bethesda, MD, USA).

#### Statistical analysis

Results are reported as the mean ± S.D. Biochemical assay results were evaluated using one-way ANOVA followed by Tukey’s multiple comparisons test. GraphPad Prism version 9.3.1 (GraphPad Software, San Diego, CA) was utilized for all statistical analyses. Inflammation scores from histopathological samples are expressed using the Kruskal–Wallis test for non-parametric one-way analysis of variance, followed by Dunn’s multiple comparison test. A p-value of < 0.05 was considered indicative of statistical significance across all group comparisons.

## Results

### PZQ co-treatment mitigates LF-induced nephrotoxicity

LF-induced nephrotoxicity caused a 5.5-fold increase in serum creatinine and a 2.1-fold increase in BUN, while albumin levels decreased by 27.3% compared to the control group. Co-treatment with PZQ significantly improved these parameters, reducing serum creatinine and BUN by 71.6% and 34.2%, respectively, and increasing albumin levels 1.2-fold relative to the LF group. However, compared to controls, serum creatinine and BUN remained elevated by 1.56- and 1.4-fold, respectively, and albumin was still reduced by 13% **(**Table 2**)**.

**Table 2:** The effect of LF (10 mg/kg/48 h, p.o.) and PZQ (300 mg/kg/day, p.o) co-treatment for 8 weeks on serum creatinine, blood urea nitrogen, and albumin

Groups	Serum creatinine (mg/dl)	BUN (mg/dl)	Albumin (g/dl)
Control	0.16 ± 0.01	32.52 ± 1.18	3.8 ± 0.23
LF	0.88 ± 0.01^a^	69.15 ± 3.73^a^	2.77 ± 0.14^a^
LF + PZQ	0.25 ± 0.02^ab^	45.48 ± 1.49^ab^	3.31 ± 0.18^ab^
PZQ	0.15 ± 0.01^b^	34.2 ± 2.17^b^	3.68 ± 0.22^b^

Mean ± S.D. (*n* = 6) is how the data are presented. a, b: Statistically distinct from the control and LF groups, respectively, at P < 0.05, as determined by ANOVA with subsequent Tukey–Kramer post-hoc analysis

*LF* Leflunomide, *PZQ* Praziquantel, *BUN* Blood Urea Nitrogen

### PZQ co-treatment effect on histopathological changes

**Fig. 1:**
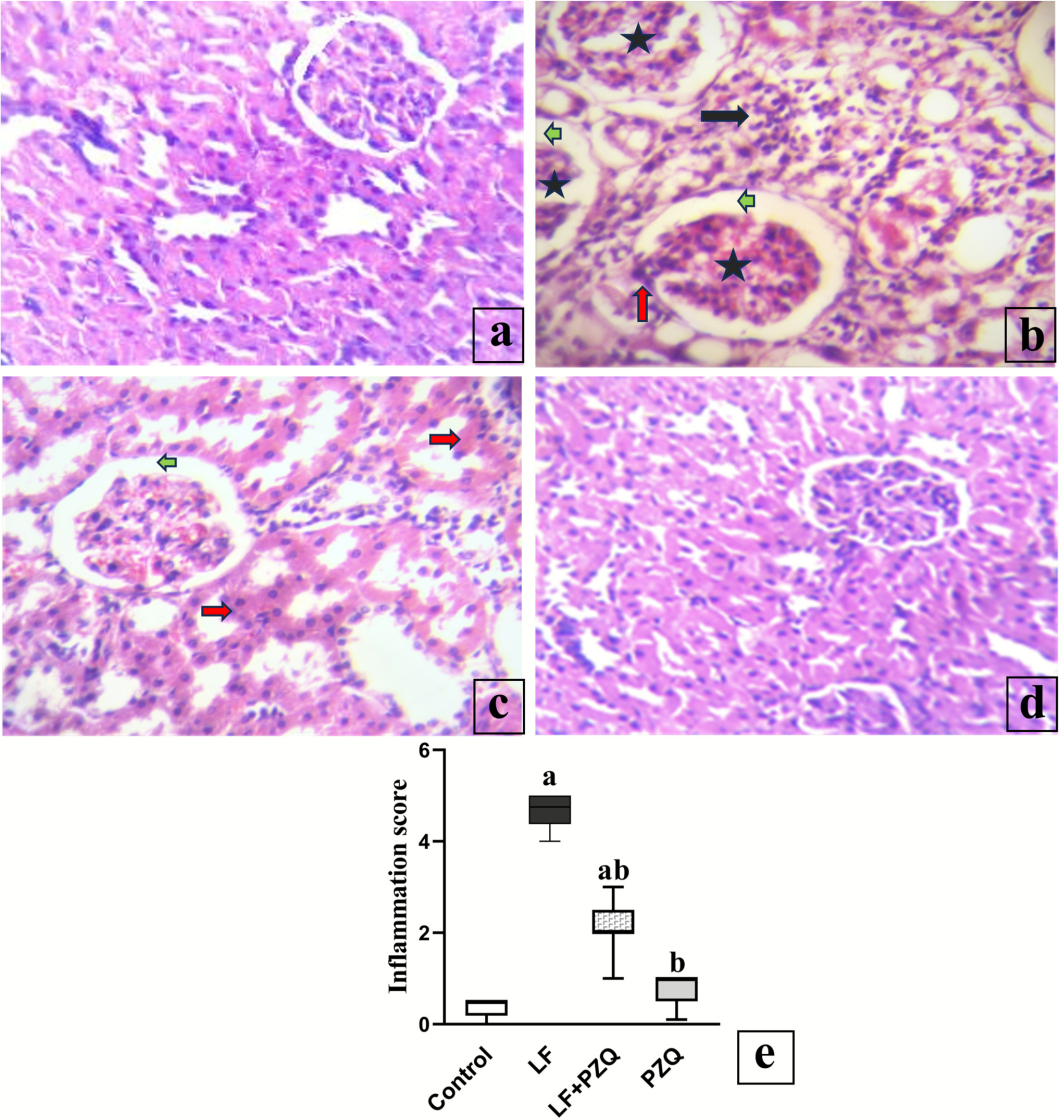
Representative histopathological examination of renal tissue sections stained with hematoxylin and eosin (X400). (**a** and **d**) Kidney tissues from the control and PZQ-only groups show typical kidney structure. (**b**) Kidney tissues from the LF group showing glomerular atrophy ***(stars)*** with increased capsular space ***(green arrows)***, glomeruli adherent to Bowman’s capsule ***(red arrow***), severe degenerative changes, necrosis of tubular epithelia, and infiltration by massive leukocytes ***(black arrow).*** (**c**) Kidney tissues from mice receiving LF and cotreated with PZQ showing a slight increase in Bowman’s capsule ***(green arrow),*** with hydropic degeneration of tubular epithelium ***(red arrows).* (e)** Inflammation was scored based on the following criteria: ≤ 0.5 indicates no inflammation, 0.5–1 indicates mild inflammation, 2–3 indicates moderate inflammation, and 4–5 indicates severe inflammation. A Kruskal–Wallis one-way analysis of variance on ranks was used, with Dunn’s multiple comparisons test to identify group differences. Significance was defined as *p* < 0.05. (a) refers to a statistically significant difference compared with the control group, and (b) points to a significant difference from the LF group

In control mice, the renal corpuscle consists of the glomerulus and Bowman’s capsule. Bowman’s capsule is incomplete at the urinary pole, with simple squamous epithelium transitioning to cuboidal epithelium in the proximal convoluted tubules **(**Fig. 1a**)**. Similarly, kidneys from the PZQ-only treated group displayed typical structural arrangements consistent with the control **(**Fig. 1d**)**. However, in the LF group, the kidney cortex exhibited glomerular atrophy, characterized by shrinkage of the glomerular tuft and increased capsular space. In some cases, the glomeruli showed marked hypercellularity due to endothelial cell proliferation, accompanied by diffuse eosinophilic sclerosis that obliterated Bowman’s capsule and capillary lumens, with areas of adhesion to a fibrosed Bowman’s capsule. Renal tubules displayed loss of brush borders, degenerative changes progressing to necrosis in some cases, and leukocyte infiltration. **(**Fig. 1** b)**.

Meanwhile, the kidney of the LF/PZQ group displays standard renal tissue architecture. A slight increase in Bowman’s capsule size and congestion of glomerular capillaries and intertubular blood vessels are observed in some instances. Additionally, there is mild hydropic degeneration of regenerated tubular epithelium with a loss of brush borders **(**Fig. 1c**)**.

### PZQ co-treatment alleviates LF oxidative stress

**Fig. 2:**
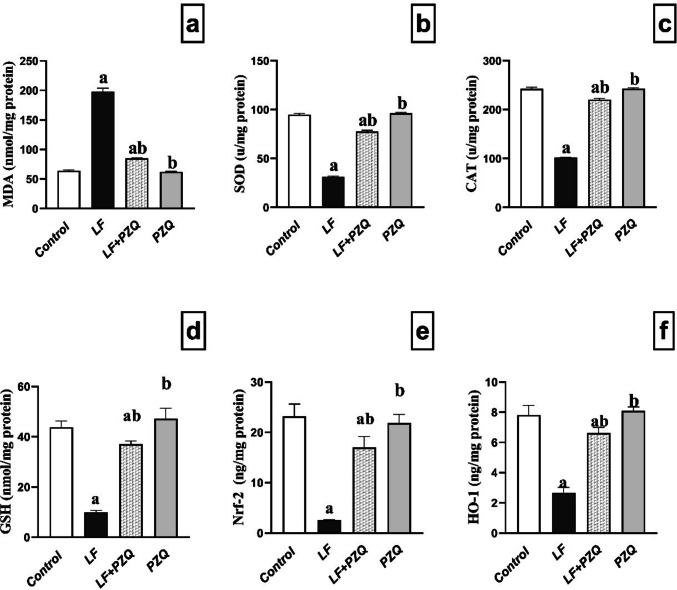
Effect of LF (10 mg/kg/48 h, p.o.) and PZQ (300 mg/kg/day, p.o.) co-treatment for 8 weeks on the oxidative stress markers. (**a**) *MDA* levels in mice administered LF alone and in combination with PZQ. (**b**) *SOD* levels in mice receiving LF, with or without PZQ co*-*administration. (**c**) *CAT* levels in mice administered LF alone and in combination with PZQ. (**d**) *GSH* levels in mice receiving LF, with or without PZQ co-administration. (**e**) *Nrf-2* levels in mice administered LF alone and in combination with PZQ. (**f**) *HO-1* levels in mice receiving LF, with or without PZQ co-administration. Results are expressed as mean ± standard deviation (*n* = 6). (a) signifies a statistically significant difference relative to the control group, and (b) indicates a significant difference compared to the LF group. Statistical analysis was conducted using one-way ANOVA, followed by the Tukey–Kramer post hoc test. A *p*-value of less than 0.05 was considered statistically significant

Assessment of oxidative stress revealed that renal *MDA* levels were elevated 3.1-fold in the LF group compared to the control. Co-treatment with PZQ significantly reduced *MDA* by 56.98% relative to the LF group, although levels remained 1.33-fold higher than in controls. In the LF group, antioxidant markers *SOD, CAT, GSH, Nrf-2,* and *HO-1* were markedly decreased by 66.9%, 58.0%, 77.3%, 89.1%, and 66.1%, respectively, compared to controls. The LF-PZQ group substantially restored these antioxidants, with increases of 2.5-, 2.2-, 3.7-, 6.7-, and 2.5-fold relative to the LF group, while remaining slightly below control levels (decreased by 18%, 9%, 15.5%, 26.6%, and 15.1%, respectively) **(**Fig. 2**)**.

### PZQ co-treatment improves renal inflammation

**Fig. 3:**
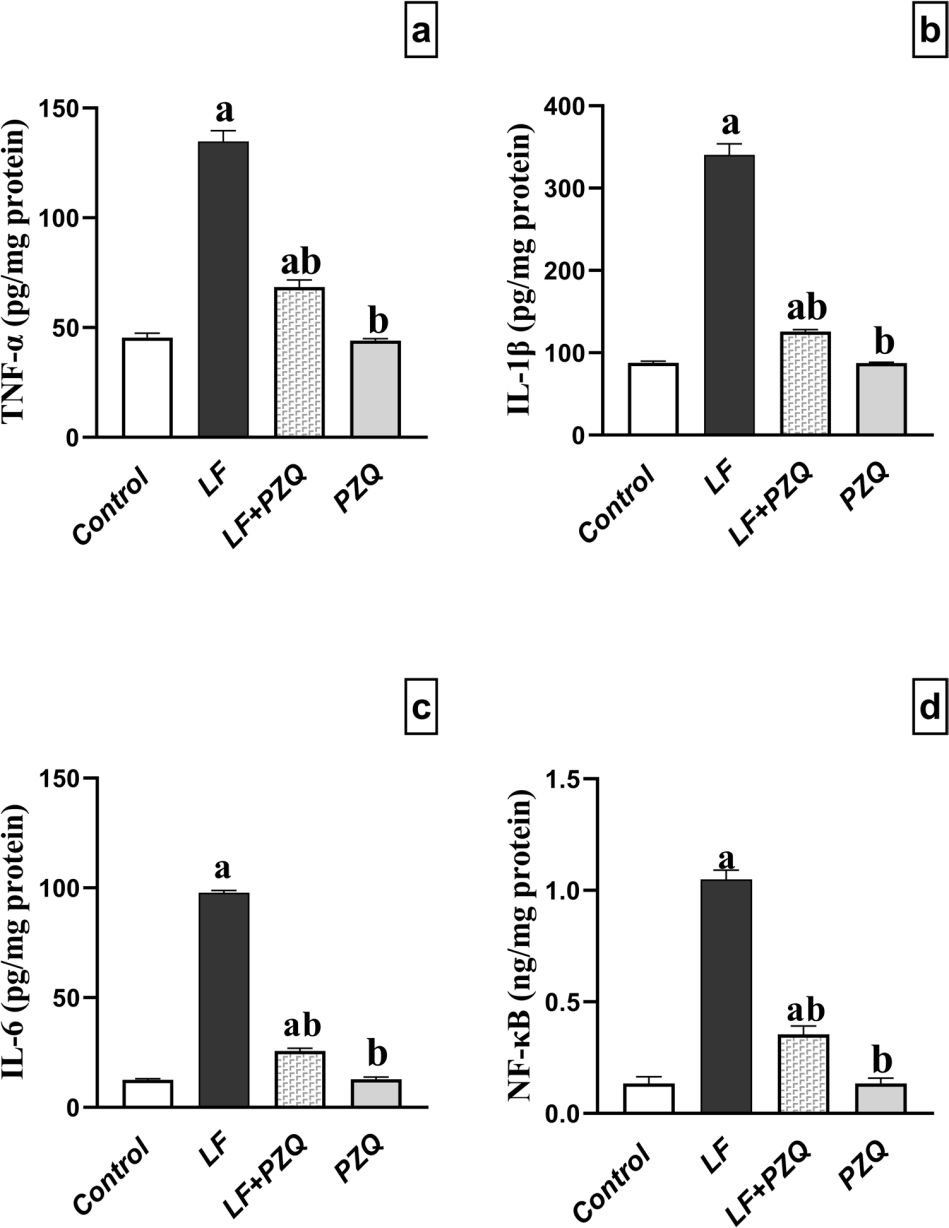
Effect of LF (10 mg/kg/48 h, p.o.) and PZQ (300 mg/kg/day, p.o.) co-treatment for 8 weeks on the inflammatory markers. (**a**) *TNF-α* levels in mice administered LF alone and in combination with PZQ. (**b**) *IL-1β* levels in mice receiving LF, with or without PZQ co-administration. (**c**) *IL-6* levels in mice administered LF alone and in combination with PZQ. (**d**) *NF-κB* levels in mice receiving LF, with or without PZQ co-administration. Results are expressed as mean ± standard deviation (*n* = 6). (a) signifies a statistically significant difference relative to the control group, and (b) indicates a significant difference compared to the LF group. Statistical analysis was conducted using one-way ANOVA, followed by the Tukey–Kramer post hoc test. A *p*-value of less than 0.05 was considered statistically significant

Inflammation is a key feature of LF-induced nephrotoxicity. LF treatment significantly elevated *TNF-α, IL-1β, IL-6,* and *NF-κB* levels by 2.97-, 3.9-, 7.8-, and 7.8-fold, respectively, compared to the control group, confirming an inflammatory response. Co-treatment with PZQ markedly reduced these levels by 49.2%, 63%, 73.7%, and 66.3%, respectively, relative to the LF group, although they remained elevated at 1.5-, 1.4-, 2-, and 2.6-fold, respectively, compared to the control **(**Fig. 3**)**.

### PZQ co-treatment effect on renal *PPAR-γ* level

**Fig. 4:**
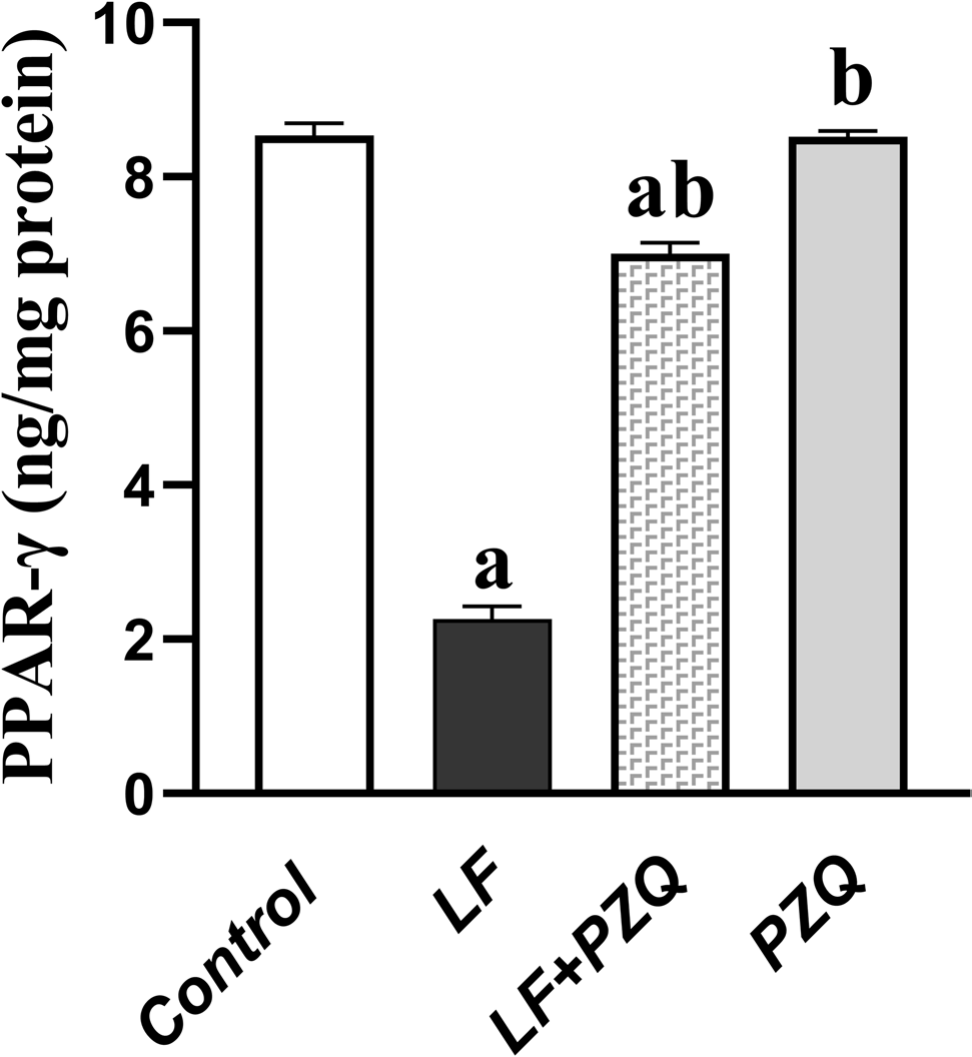
Effect of LF (10 mg/kg/48 h, p.o.) and PZQ (300 mg/kg/day, p.o.) co-treatment for 8 weeks on *PPAR-γ* expression. Data are expressed as mean ± S.D. (*n* = 6). (**a**) denotes a statistically significant difference compared to the control group, while (**b**) represents a significant difference relative to the LF group. Statistical comparisons were conducted using one-way ANOVA followed by Tukey–Kramer post hoc analysis, with a significance threshold set at *p* < 0.05. *PPAR-γ*, peroxisome proliferator-activated receptor-γ

PPAR-γ levels were markedly decreased in the LF group, showing a 73.5% reduction compared to the control. Co-treatment with PZQ significantly alleviated this suppression, increasing *PPAR-γ* levels 3.1-fold relative to the LF group, although they remained 17.95% lower than in the control group **(**Fig. 4**)**.

### PZQ co-treatment effect on Wnt/β-catenin signaling genes expression

**Fig. 5:**
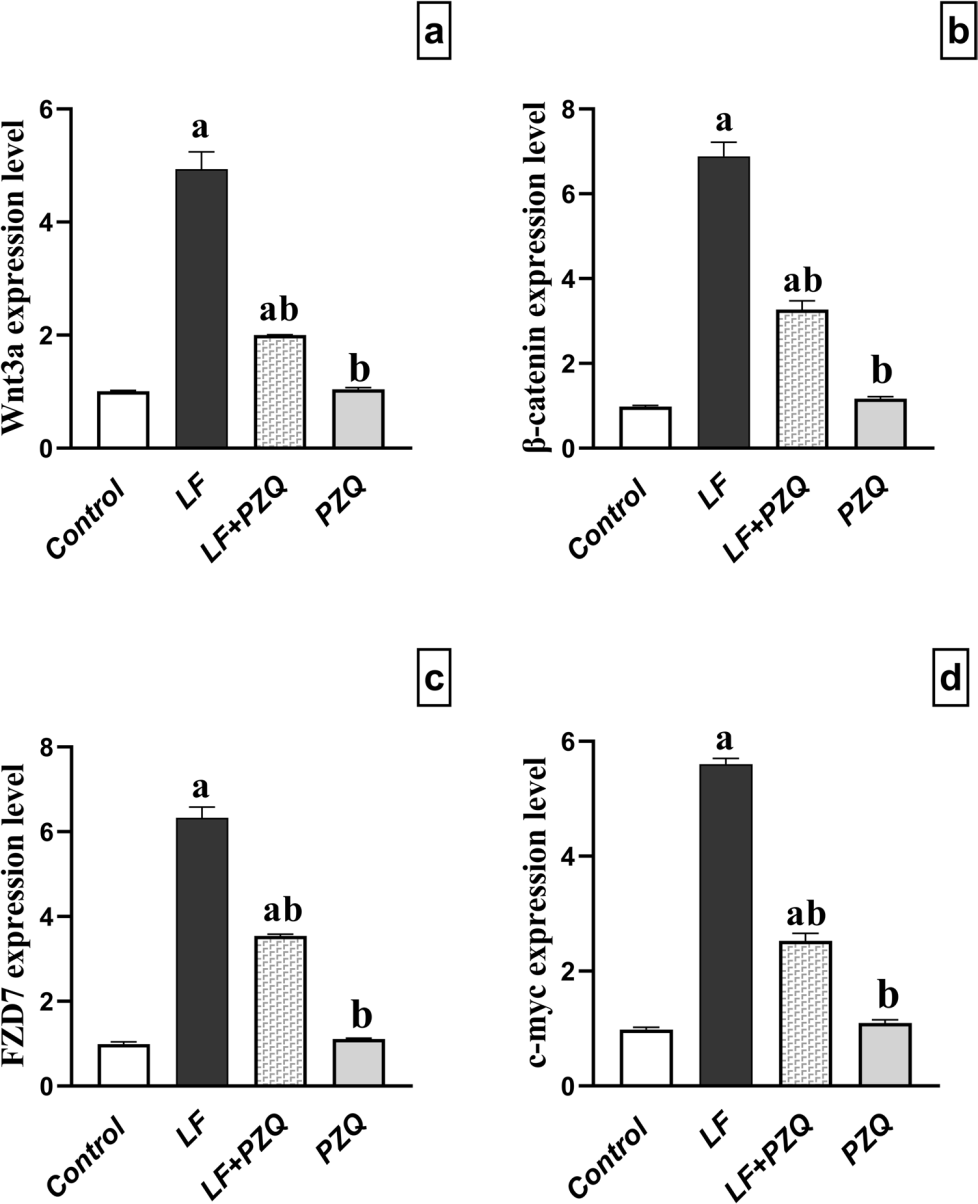
Effect of LF (10 mg/kg/48 h, p.o.) and PZQ (300 mg/kg/day, p.o.) co-treatment for 8 weeks on *Wnt3a, β-catenin, FZD7,* and *c-myc* gene expression. **(a)** Quantitative evaluation of *Wnt3a* gene expression in mice treated with LF and co-treated with PZQ. **(b)** Quantification of *β-catenin* gene expression in mice treated with LF and co-treated with PZQ. **(c)** Analysis of *FZD7* gene expression in mice treated with LF and co-treated with PZQ. **(d)** Evaluation of *c-myc* gene expression in mice treated with LF and co-treated with PZQ. Results are expressed as the mean ± S.D. (n = 3). (a) Indicates a statistically significant difference compared to the control group, while (b) Denotes a significant difference from the LF group. Statistical analysis was performed using one-way ANOVA followed by the Tukey–Kramer test, with a significance level set at p < 0.05

The Wnt/β-catenin signaling pathway was examined, revealing that LF-induced nephrotoxicity significantly upregulated the expression of *Wnt3a*, *β-catenin*, *FZD7*, and *c-myc* by 4.9-, 7-, 6.3-, and 5.6-fold, respectively, compared to the control group. Co-treatment with PZQ markedly reduced their expression by 59.4%, 52.5%, 44.1%, and 54.8%, respectively, relative to the LF group, although levels remained elevated at 2-, 3.33-, 3.57-, and 2.57-fold, respectively, compared to the control **(**Fig. 5**).**

### PZQ co-treatment effect on TGF-β signaling genes expression

**Fig. 6:**
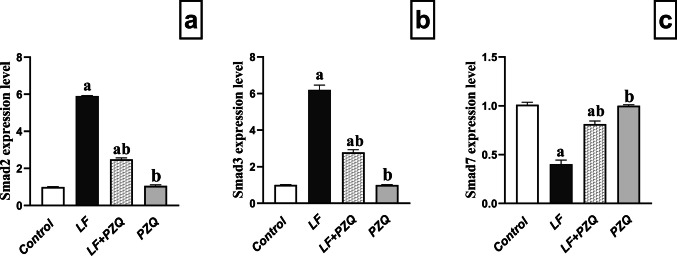
Effect of LF (10 mg/kg/48 h, p.o.) and PZQ (300 mg/kg/day, p.o.) co-treatment for 8 weeks on *Smad2, Smad3,* and *Smad7* gene expression. **(a)** Represent *Smad2* gene expression in mice treated with LF and cotreated with PZQ. **(b)** Analysis of *Smad3* gene expression in mice treated with LF and co-treated with PZQ. **(c)** Evaluation of *Smad7* gene expression in mice treated with LF and co-treated with PZQ. Data are presented as mean ± S.D. (n = 3). (a) Indicates a significant difference compared to the control group, while (b) denotes a significant difference compared to the LF group. A one-way ANOVA was applied to determine statistical significance, followed by the Tukey–Kramer test. A threshold of *p* < 0.05 indicated significant differences

The expression of *Smad2, Smad3,* and *Smad7*, key mediators of *TGF-β* signaling, was evaluated. LF treatment significantly increased *Smad2* and *Smad3* expression by 5.8-fold and 6.1-fold, respectively, while *Smad7* expression decreased by 60% compared to the control group. Co-treatment with LF/PZQ reduced *Smad2* and *Smad3* expression by 57.8% and 55%, respectively, and upregulated *Smad7* approximately twofold relative to the LF-treated group. Compared with the control group, the LF/PZQ group still showed moderate increases in *Smad2* and *Smad3* (2.4-fold and 2.8-fold, respectively) and a slight decrease in *Smad7* expression (19.5%) **(**Fig. 6**)**.

**Fig. 7:**
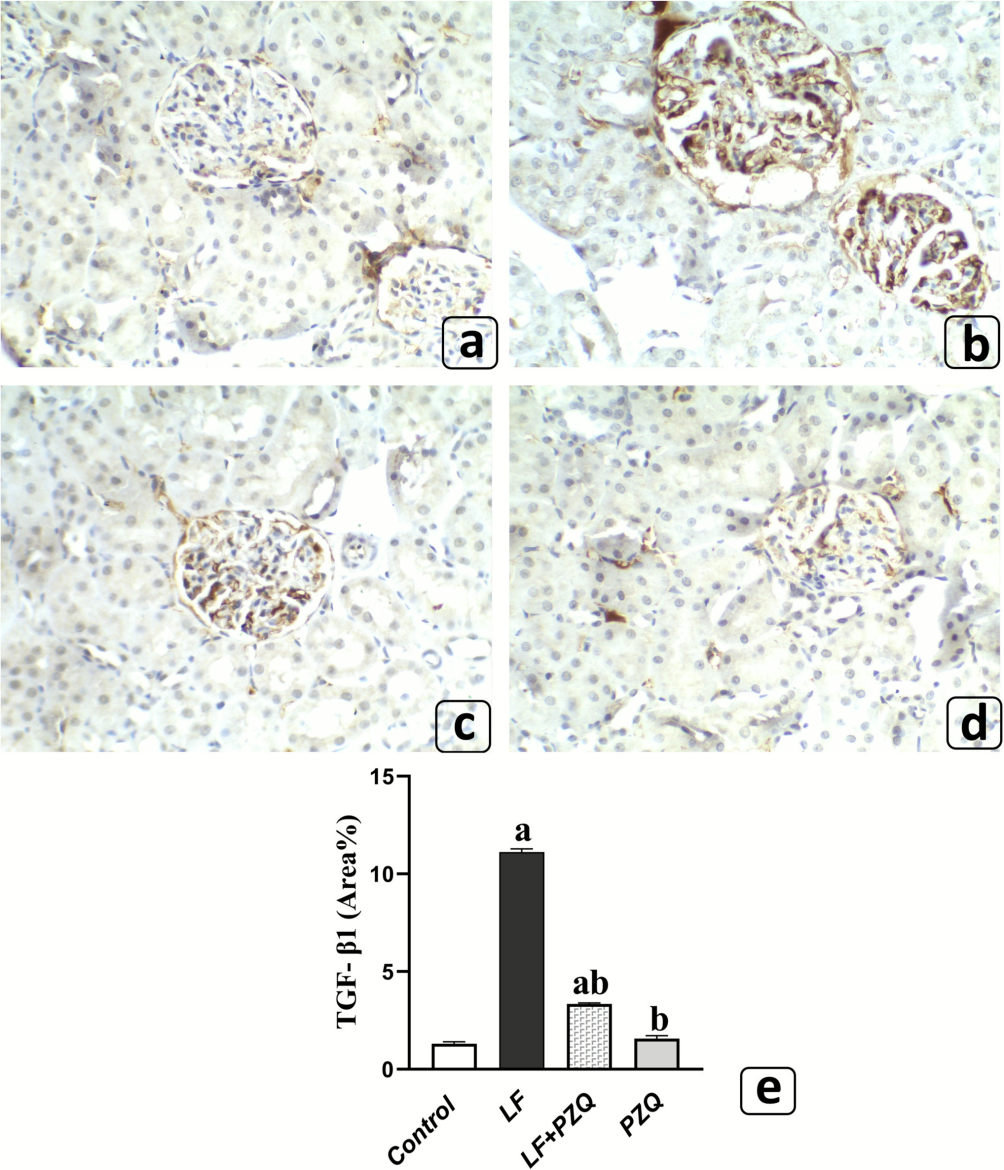
Effect of LF (10 mg/kg/48 h, p.o.) and PZQ (300 mg/kg/day, p.o.) co-treatment for 8 weeks on *TGF-β1* expression. Based on Immunohistochemical Staining. (**a**, **d**) Kidney tissues from control and PZQ-only groups show low *TGF*-*β1* expression in renal tissue. (**b**) Kidney tissues from the LF group exhibit severe *TGF-β1* expression (brown staining). (**c**) Kidney tissues from the LF/PZQ-treated group display mild *TGF-β1* expression (brown staining). All sections were observed at a magnification of X400. (**e**) Quantitative image analysis is expressed as the region’s percentage with an immunopositive reaction. Data are presented as mean ± S.D. (*n* = 4). (a) Indicates significant differences compared to the control group, and (b) indicates significant differences compared to the LF group. The study employed a one-way ANOVA for data analysis, with the Tukey–Kramer test used for post hoc comparisons. Statistical significance was set at *p* < 0.05

In parallel, *TGF-β1* expression was markedly elevated, showing an 8.5-fold increase in the LF group compared to the control. Co-treatment with PZQ significantly reduced this rise by 69.9% relative to the LF group, although *TGF-β1* levels remained 2.6-fold higher than in the control group **(**Fig. 7**)**.

## Discussion

The current work studied the possible nephroprotective role of PZQ against LF-induced nephrotoxicity in a mouse model. This result is confirmed by various reported effects, including the inhibition of the TGF-β/Smad/Wnt/β-catenin/NF-κB signaling pathway, a decrease in inflammatory markers (TNF α, IL-6, NF-κB, and IL-1β), mitigation of oxidative stress, and an elevation in the levels of the anti-inflammatory, anti-oxidant PPAR-γ and Nrf-2.

The immunosuppressant LF is linked to significant unfavorable effects affecting the hepatic, immunological, hematological, and respiratory systems (El-Sherbiny et al. 2021). The nephrotoxicity of LF has been reported in several case studies showing kidney dysfunction and severe interstitial nephritis as significant adverse effects(Nakafero et al. 2021; Hurtado Uriarte et al. 2016; Pinto et al. 2013). Aljohani et al. confirmed LF’s nephrotoxicity in mice at the recommended therapeutic levels, especially at 10 mg/kg (Aljohani et al. 2022). From this point, the current study explored the cellular mechanisms of LF-induced nephrotoxicity.

Impairment of renal function is a significant indicator of drug-induced nephrotoxicity (Kim and Moon 2012). The current investigation findings demonstrated that LF significantly impaired renal function, as evidenced by increased serum creatinine and BUN levels and decreased albumin levels. These results align with an earlier study, where LF caused a significant impairment of kidney function (Aljohani et al. 2022). On the contrary, as anticipated, PZQ appears to mitigate LF-induced nephrotoxicity, as indicated by improved renal function.

Leflunomide-induced nephrotoxicity was further confirmed through histopathological examination, which revealed glomerular atrophy, increased capsular space, and a significant increase in cellularity due to endothelial cell proliferation. Degenerative changes were observed in the renal tubules, progressing to widespread necrosis, in the LF group. These findings are consistent with those reported in previous studies (Aljohani et al. 2022). PZQ mitigated these histopathological changes, as evidenced by a marked reduction in pathological alterations, with only mild tubular degeneration and occasional congested blood vessels observed in some cases.

Elevated kidney marker levels and decreased anti-oxidant levels due to oxidative damage are critical indicators of nephrotoxicity (Kim and Moon 2012). In the same context, numerous studies have indicated increased oxidative stress in kidney disease, which is closely linked to drug-induced nephrotoxicity (Azırak and Özgöçmen 2023; Piko et al. 2023). Previous studies have demonstrated that Nrf-2, known for its anti-oxidant and anti-inflammatory properties, can alleviate oxidative stress by neutralizing reactive oxygen species (ROS) and promoting the synthesis of anti-oxidant and anti-inflammatory enzymes (Satta et al. 2017). In the current study, administration of LF severely disrupted redox equilibrium, as reflected by a substantial rise in MDA levels and a marked decline in CAT, GSH, SOD, HO-1, and Nrf-2 levels. These results correspond with a prior study, demonstrating that LF causes oxidative damage in a liver model (Lodhi et al. 2013). On the contrary, in the current study, PZQ exhibited notable anti-oxidant effects, evidenced by a significant increase in Nrf-2 activity and enhanced anti-oxidant enzyme levels (SOD, GSH, and CAT and HO-1).

Additionally, PZQ treatment was associated with reduced MDA concentrations. Collectively, the observed elevation in Nrf-2 and anti-oxidant enzyme activities following PZQ administration underscores its protective role against nephrotoxicity induced by oxidative stress. This finding is consistent with earlier studies on hepatic and muscular tissues in fish models (Zuskova et al. 2018).

Oxidative stress and inflammation are intrinsically connected pathophysiological phenomena (Biswas 2016). The development of nephrotoxicity is heavily influenced by inflammation. In the present study, elevated kidney levels of TNF-α, IL-6, NF-κB and IL-1β showed a significant pro-inflammatory response in the LF group. These results support earlier studies suggesting that chronic use of LF induces an inflammatory response in liver and lung models (Elshaer et al. 2019; El-Sherbiny et al. 2021). Previous studies identified LF as an immunosuppressant and anti-inflammatory agent, attributing its effects to TNF-α downregulation. However, another study suggests this does not contradict our findings, proposing that chronic LF use may lead to inflammatory and cytotoxic effects (Elshaer et al. 2019).

Co-administration of PZQ promoted a significant reduction in the levels of renal inflammatory mediators, possibly due to its ability to suppress NF-κB activity (Qing et al. 2016). In parallel, up-regulation of Nrf-2 expression could be another reason for decreasing IL-1β, IL-6 and TNF-α. The anti-inflammatory effect of PZQ is consistent with a previous study, which demonstrated that PZQ targets Smad7, inhibiting the NLRP3 inflammasome in Schistosoma japonicum Infection (Kong et al. 2019).

Progressive nephrotoxicity is a complex process influenced by various factors, including growth factors, cytokines, metabolic pollutants, and stress-related molecules. One of the primary mediators in this pathogenesis is TGF-β1 (Gu et al. 2020). The present investigation revealed that LF markedly elevated kidney TGF-β and Smad2/3 complex levels while markedly suppressing Smad7, signifying persistent stimulation of the TGF-β/Smad signaling pathway and leading to nephrotoxicity. This aligns with Previous studies that have reported that LF significantly increases renal and hepatic TGF-β levels in mice (Elshaer et al. 2019; Aljohani et al. 2022). TGF-β is a multifunctional cytokine that controls a diverse array of cellular functions, such as growth, differentiation, apoptosis, wound repair, and the development of fibrosis (Chen et al. 2018). The TGF-β/Smad signaling pathway is initiated when TGF-β binds to TβR2, which then recruits TβR1 to activate Smad2/3. The Smad2/3 complex binds with Smad4 and migrates to the nucleus to commence the transcription of specific genes. Smad3 facilitates renal fibrosis and inflammation, whereas Smad7 has renoprotective benefits by obstructing Smad3’s interaction with active TβR1, thus diminishing TGF-β/Smad signaling (Lan 2011; Troncone 2018). From this point, nephrotoxicity arises from the imbalance between the activation of pro-fibrotic Smad3 and the suppression of antifibrotic Smad7 (Meng et al. 2016). Interestingly, co-treatment with PZQ resulted in a notable reduction in the expression of TGF-β and Smad2/3 and a significant increase in Smad7 in the LF/PZQ group. These findings are consistent with earlier investigations in which PZQ exhibited antifibrotic effects by increasing Smad7 expression in a liver fibrosis model. The increased Smad7 expression inhibited Smad2/3 phosphorylation, suppressing TGF-β/Smad signaling and mitigating fibrosis(Liu et al. 2019).

This study revealed that LF markedly enhanced renal Wnt/β-catenin signaling and the expression of its target gene c-myc. Wnt/β-catenin signaling, essential for embryonic development and physiological growth, is reactivated in response to kidney injury. While transient activation facilitates repair and regeneration, sustained and uncontrolled activation contributes to renal fibrosis. In the canonical Wnt signaling pathway, β-catenin is typically phosphorylated by the destruction complex, leading to its ubiquitination by E3 ubiquitin ligase β-TrCP and degradation by proteasomes. However, when Wnt ligands bind to FZD receptors and LRP5/6, they recruit DVL protein, which inhibits the destruction complex. This disrupts β-catenin degradation, enabling it to accumulate and translocate to the nucleus. In the nucleus, β-catenin binds to TCF/LEF co-transcription factors, stimulating the expression of Wnt target genes, such as c-myc (Schunk et al. 2021). The versatile transcription factor c-myc governs critical cellular activities, including proliferation, differentiation, and apoptosis. The overexpression of c-myc exacerbates renal fibrosis by activating the TGF-β pathway (Zhou et al. 2020; Shen et al. 2017).

The cross-talk linking the Wnt/β-catenin and TGF-β/Smad pathways was previously demonstrated in a chondrocyte model, revealing that Smad3 associates with the E3 ubiquitin ligase β-TrCP, thereby inhibiting β-catenin ubiquitination and indirectly enhancing the Wnt/β-catenin pathway (Dao et al. 2007; Li et al. 2006). Conversely, PZQ significantly diminished the expression of Wnt/β-catenin signaling and its target gene c-myc, thereby providing evidence for the interaction linking the Wnt/β-catenin and TGF-β/Smad signaling pathways. In the same context, Wnt/β-catenin signaling and NF-κB are linked through positive cross-talk. Activating the Wnt/β-catenin pathway enhances NF-κB activity, whereas nuclear β-catenin binds to NF- κB p65 subunit and increases the transcription activity of NF-κB, and increases the inflammation process (Kim et al. 2024). From this point, activation of TGF-β signaling stimulates the Wnt/β-catenin pathway by promoting β-catenin nuclear translocation. This activation enhances cross-talk with the NF-κB signaling cascade. As a result, pro-inflammatory and fibrotic gene expression is increased. Consequently, renal fibrosis and inflammation are intensified, and disease progression is promoted.

In parallel, in this study, LF resulted in a significant decrease in renal PPAR-γ expression, attributed to the overexpression of TGF-β. This finding aligns with previous studies demonstrating TGF-β-induced downregulation of PPAR-γ expression in lung fibrosis models (Lakshmi et al. 2017). PPAR-γ is a ligand-activated transcription factor crucial for controlling glucose and lipid metabolism, and also plays a significant role in regulating immune function and inflammatory responses (Vallée and Lecarpentier 2018). PPAR-γ agonists activate Smad7, inhibiting TGF-β signaling and promoting their antifibrotic properties (Vallée et al. 2017). Conversely, TGF-β1 drives myofibroblast differentiation by suppressing PPAR-γ, both directly at the transcriptional level through a Smad repressor complex that targets the TGF‐β inhibitory element and to Smad‐binding elements sequences in the PPAR-γ promoter and indirectly by increasing β-catenin levels (Lakshmi et al. 2017; Vallée and Lecarpentier 2018), respectively. These opposing biological effects draw attention to the intricate balance and complex cross-talk between PPAR γ and TGF-β. In contrast, PZQ resulted in a marked increase in renal PPAR-γ levels by reducing TGF-β levels. This also supports the potential anti-oxidant and anti-inflammatory effects of PZQ, as PPAR-γ is known to exert such effects by reducing oxidative stress-induced MDA formation and lowering pro-inflammatory cytokine levels (Muzio et al. 2021; Kim and Yang 2013).

Furthermore, the Wnt/β-catenin pathway and PPAR-γ are linked through negative cross-talk. Suppressing the Wnt/β-catenin pathway enhances PPAR-γ activity, whereas activating PPAR-γ leads to decreased β-catenin levels across several cellular systems (Vallée and Lecarpentier 2018). PPAR-γ agonists stimulate DKK and Smad7, inhibiting the Wnt/β-catenin pathway and facilitating β-catenin degradation via the proteasome degradation. In contrast, Wnt ligands suppress GSK-3β, resulting in the cytosolic accumulation of β-catenin. Accumulated β-catenin migrates into the nucleus, interacting with TCF/LEF transcription factors, ultimately inhibiting PPAR-γ expression (Vallée et al. 2017).

**Fig. 8:**
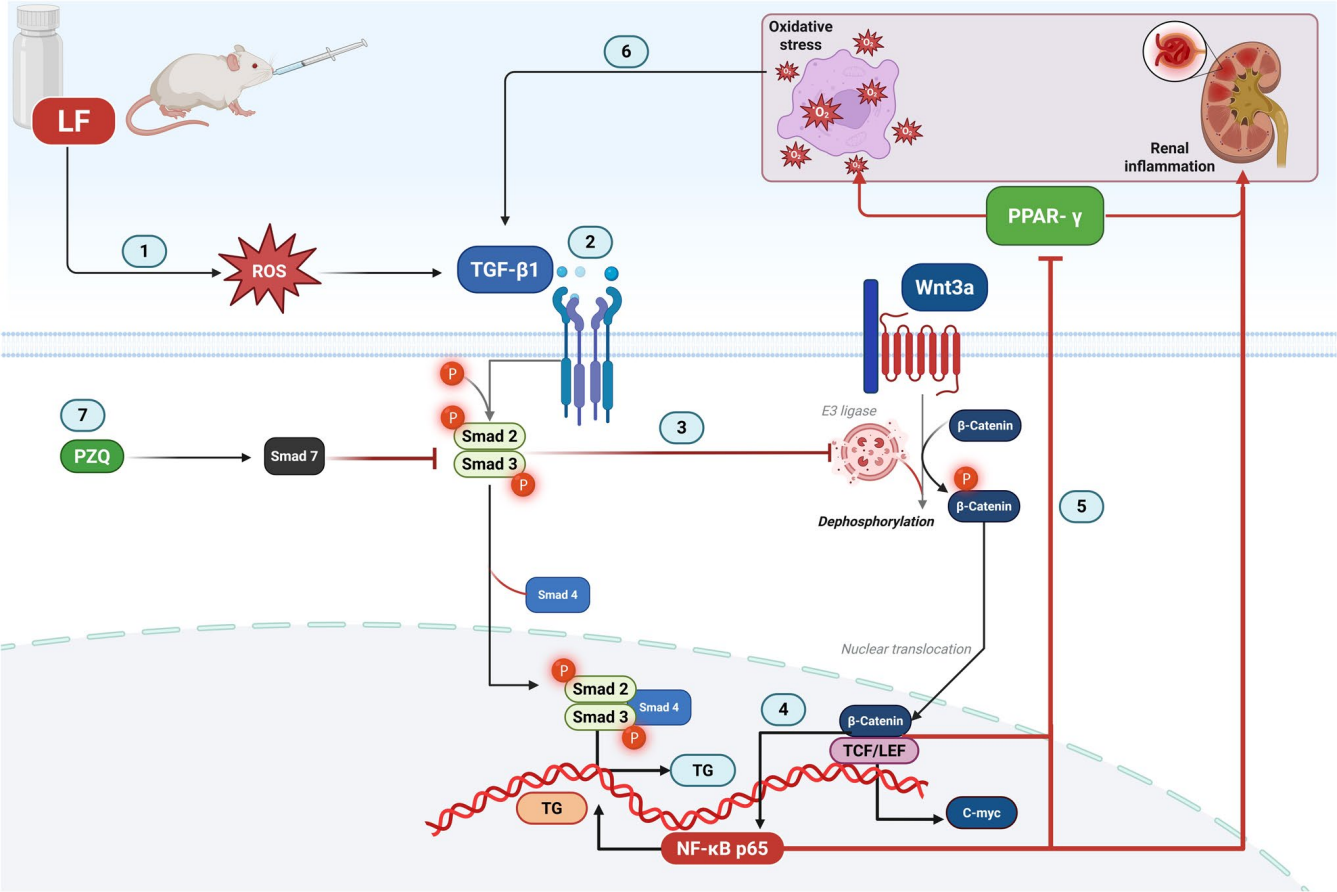
The mechansistic figure illustrating the crosstalk between TGF-β/Smad, Wnt/β-catenin, NF-κB, and PPAR-γ signaling, and the modulating effects of PZQ. (1) indicates that LF increases oxidative stress, leading to overexpression of TGF-β, which activates the TGF-β/Smad signaling pathway. (2) shows that upon TGF-β binding to TβR2, TβR1 is recruited, resulting in Smad2/3 activation. The Smad2/3 complex is associated with Smad4 and translocates to the nucleus to initiate transcription of target genes. (3) illustrates Smad3 interaction with the E3 ubiquitin ligase β-TrCP, inhibiting β-catenin ubiquitination and thereby enhancing Wnt/β-catenin signaling, demonstrating the crosstalk between TGF-β/Smad and Wnt/β-catenin pathways. (4) shows the positive regulation of β-catenin to NF-κB. (5) shows nuclear translocation of β-catenin, which binds to TCF/LEF transcription factors, suppressing PPAR-γ expression. (6) indicates that activation of TGF-β/Smad and Wnt/β-catenin signaling, along with PPAR-γ inhibition, exacerbates oxidative stress and inflammation, further amplifying TGF-β signaling and pathway crosstalk. (7) highlights PZQ-mediated activation of Smad7, which inhibits Smad2/3 phosphorylation, thereby attenuating TGF-β/Smad signaling and its associated crosstalk

Collectively, our study depicts how Activation of TGF-β signaling leads to the stimulation of the canonical Wnt/β-catenin pathway through several mechanisms, including the suppression of Wnt antagonists (E3 ubiquitin ligase β-TrCP) and the promotion of β-catenin nuclear translocation. Once activated, Wnt/β-catenin signaling causes two major downstream effects. First, it suppresses the expression of PPARγ, which in turn increases oxidative stress, inflammation, and fibrotic processes within the kidney tissue. Second, it simultaneously activates NF-κB signaling, thereby intensifying renal inflammation. Notably, therapeutic intervention with PZQ has been shown to counteract these pathological mechanisms, alleviating both the oxidative, fibrotic and inflammatory consequences induced by the cross-talk between these signaling pathways as illustrated in Fig. 8** (the mechanistic Figure)**.

## Conclusion

This study is the first to reveal the role of TGF-β/Smad/Wnt/β-Catenin/NF-κB/Nrf-2 signaling pathway inhibition and upregulation of PPAR-γ in the nephroprotective effect of PZQ against LF-induced nephrotoxicity. Reduced renal markers levels evidence this protective effect, decreased renal oxidative stress, improved inflammatory status, downregulation of TGF-β/Smad/Wnt/β-Catenin/NF-κB signaling pathway, and increased anti-oxidant, anti-inflammatory PPAR-γ and Nrf-2 expression. Collectively, these outcomes suggest a new potential application for anthelmintic drugs in treating nephrotoxicity.

## Data Availability

All datasets used and/or analyzed during the current study will be made available by the corresponding author upon request.

## Abbreviations

LF: Leflunomide
DHODH: Dihydroorotate dehydrogenase
FZD: Frizzled
LRP5/6: Low-density lipoprotein receptor-related proteins 5 and 6
GSK-3: Glycogen synthetase kinase
Dvl: Disheveled
TCF/LEF: T cell factor/lymphoid enhancing factor
DKK: Dickkopf-1
PPAR-γ: Peroxisome proliferator-activated receptor-γ
PZQ: Praziquantel
TGF-β1: Transforming growth factor-β1
TβR2: TGF-β receptor 2
TβR1: TGF-β receptor 1
β-TrCP: Beta-transducin repeats-containing proteins
BUN: Blood urea nitrogen
MDA: Malondialdehyde
SOD: Superoxide dismutase
GSH: Glutathione
CAT: Catalase
TNF-α: Tumor necrosis factor –alpha
IL-1β: Interleukin-1β
IL-6: Interleukin-6
Nrf-2: Nuclear factor erythroid 2-related factor 2
HO-1: Heme oxygenase 1
SDS: Sodium dodecyl sulfate
HRP: Horseradish peroxidase
ECL: Luminol- based chemiluminescent
TBS: Tris-buffered saline
H&E: Hematoxylin and eosin
ANOVA: One-way analysis of variance
ELISA: Enzyme-linked immunosorbent assay
